# Revisiting Estimates of CTL Killing Rates *In Vivo*

**DOI:** 10.1371/journal.pone.0001301

**Published:** 2007-12-12

**Authors:** Andrew Yates, Frederik Graw, Daniel L. Barber, Rafi Ahmed, Roland R. Regoes, Rustom Antia

**Affiliations:** 1 Department of Biology, Emory University, Atlanta, Georgia, United States of America; 2 Institute of Integrative Biology, Eidgenössische Technische Hochschule (ETH) Zurich, Zurich, Switzerland; 3 National Institute of Allergy and Infectious Diseases (NIAID), Bethesda, Maryland, United States of America; 4 Emory Vaccine Center, Emory University School of Medicine, Atlanta, Georgia, United States of America; Federal University of São Paulo, Brazil

## Abstract

Recent experimental advances have allowed the estimation of the *in vivo* rates of killing of infected target cells by cytotoxic T lymphocytes (CTL). We present several refinements to a method applied previously to quantify killing of targets in the spleen using a dynamical model. We reanalyse data previously used to estimate killing rates of CTL specific for two epitopes of lymphocytic choriomeningitis virus (LCMV) in mice and show that, contrary to previous estimates the “killing rate” of effector CTL is approximately twice that of memory CTL. Further, our method allows the fits to be visualized, and reveals one potentially interesting discrepancy between fits and data. We discuss extensions to the basic CTL killing model to explain this discrepancy and propose experimental tests to distinguish between them.

## Introduction

A detailed understanding of immune responses requires quantifying the population dynamics of pathogens and immune cells. A key element of these dynamics is the killing of infected cells by cytotoxic T lymphocytes (CTL). We want to be able to accurately measure how fast CTL can find and kill infected target cells *in vivo*. This information will help answer many basic immunological questions. For example, how do killing rates differ between effector and memory CTL, and do these rates change as CTL become exhausted in chronic infection? Answering these questions will be particularly important for persistent infections such as HIV.

Many studies have quantified cytotoxic T cell activity *in vitro* [1]–[8]. More recently, mathematical models in conjuction with advances in experimental techniques have allowed estimates of CTL killing rates *in vivo* [9]–[14]. For a summary of the work in both of these areas, we refer the reader to our review [15].

**Figure 1:**
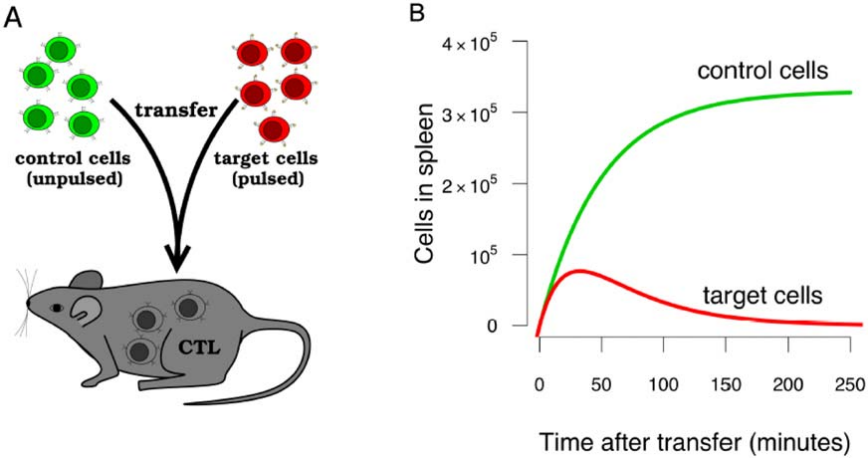
The CTL killing assay. Peptide-pulsed target cells and control (unpulsed) cells are injected intravenously (A). The control cells allow us to measure the flux of both populations into the spleen, and the differences between numbers of pulsed and unpulsed cells in the spleen at later timepoints (panel B) is assumed to be due to killing by spleen-resident CTL.

In this paper we present a refined analysis of a dataset relating to CTL killing of LCMV-infected cells in a mouse model, first presented by Barber *et al.* [16] and subsequently analysed quantitatively by some of us [13]. Barchet *et al.* [17] were among the first to use this simple but powerful assay. Briefly, effector or memory CTL are generated by infecting mice with LCMV and waiting either 8 or >30 days respectively. To measure the rate of killing of target cells by CTL, a mixture of fluorescently labelled cells containing equal proportions of unpulsed controls and cells pulsed with either of the two immunodominant LCMV epitopes (NP396 and GP276) is injected intravenously into mice. To study effector and memory CTL responses to these targets, the frequencies of CTL, control cells and pulsed targets are measured in the spleen following sacrifice of mice during the first few hours after injection of targets (Figure 1).

To estimate killing rates from this assay, we need to take into account two complications. First cells are flowing into the spleen while killing is taking place. This results in some target cells being under CTL surveillance for shorter durations than others. This was addressed in [13] using a simple dynamical model of migration and killing of target cells. They also took into account a second problem, namely that different mice have different numbers of splenocytes and spleen-resident CTL.

There are, however, further problems with this method, which we address here. The most significant of these is the uncertainty in the ‘take’ of injected cells (*i.e.* the number of injected cells that end up in the spleen). This is variable for a combination of reasons; different numbers of cells in each inoculum and difficulties associated with targeting injections precisely into the tail vein.

We show how this uncertainty can be removed from the calculation by using the pairing of the estimates of unpulsed and pulsed target cell frequencies in each animal. Importantly, this new approach also allows clearer visualisation of the fits and the data. Applying the method to the dataset first presented in [16] more than doubles previous estimates of effector CTL killing rates. Furthermore, the clearer visualization allowed by the new approach reveals a systematic discrepancy between the data and the model in one case. We propose several hypotheses to account for this shortcoming of the current framework, and we describe additional experiments that may allow us to discriminate between these hypotheses.

## Methods

### The basic model–the dynamics of target cells in blood and spleen

We begin by describing the original method of estimation, and then explain how it can be improved.

The flux of control or unpulsed cells from the blood to spleen immediately following tail-vein injection is modelled as follows. If *N* denotes the number of unpulsed cells in blood and *U* the number of unpulsed cells in the spleen, then
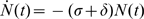
(1)

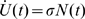
(2)or
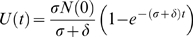
(3)where *N*(0) is the number of control cells injected into the blood at the start of the experiment. We assume that the influx of pulsed and unpulsed targets into the spleen is identical, but that in addition pulsed targets are killed in the spleen. The total rate of killing is assumed to take a mass-action form, proportional to the product of the dimensionless frequencies of target cells and CTL in the spleen. Assuming all mice have identical total splenocyte numbers , then,
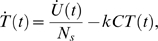
(4)where *T*(*t*) and *C* are the frequencies of target cells and CTL in the spleen, respectively. This equation assumes equal numbers of pulsed and unpulsed target cells in the inoculum.

Ideally, all mice would be identical, with the same inoculum *N*(0), spleen size *N_s_* and CTL frequency *C*, and would differ only in the time of sacrifice. If this were the case, we would estimate *k* as follows. First fit the data for unpulsed target cells (Figure 1, green line) to obtain the parameters governing the dynamics of unpulsed cells. Then use these parameters to estimate *k* by fitting equation (4) to a time series of measured target cell frequencies (Figure 1, red line).

### Biological and Experimental Limitations

The above approach needs modification, because although we would prefer all mice to be identical, in reality they differ substantially in the following:

1. Spleen sizes (splenocyte numbers);
2. Number of CTL specific for GP and NP epitopes–this depends on their response to the LCMV immunization;
3. The inocula for different mice contain slightly different proportions of pulsed and unpulsed target cells.

We took these sources of variation into account in our original study [13] by measuring *N_s_* and *C* for each animal, as well as the ratio of unpulsed to pulsed targets, *f* (measured separately for NP- and GP-pulsed targets). Then for animal *i* sacrificed at time *t*,
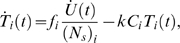
(5)where *k* is the rate constant for killing, *N_s_* is the total number of splenocytes, *U*(*t*) is the total number of unpulsed targets in the spleen and *C* is the frequency of CTL in the spleen (numbers/total splenocytes) specific for the target cells of interest.

In our original approach we fitted the following equation to the unpulsed cell data pooled from all animals at all timepoints to obtain averaged estimates of *c* = *σN*(0) and *d* = *σ*+*δ*:
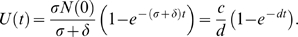
(6)This follows from equation (3). Using this in equation (5),
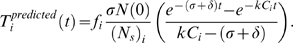
(7)The killing rate *k* was then estimated by fitting eqn. (7) to the measured target cell frequencies *T_i_*(*t*) in the spleen by minimising the quantity
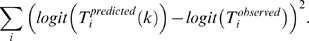
The sum is over all animals *i* and the logit transform was used to normalise the distributions of frequency measurements.

### Improving the estimate of CTL killing rate *k*

The method above uses an averaged estimate for the unpulsed cell numbers *U*(*t*) at each timepoint. This discards information, however, since the data comprise paired measurements of *T_i_*(*t*) and *U_i_*(*t*) for each animal. Using this paired information improves the fitting procedure since a further source of variation is removed–the initial ‘take’ of injected cells (*i.e.* the proportion of the injected targets that migrate to the spleen, σ/(σ+δ)) Since we expect the uncertainty in the take to be identical for pulsed and unpulsed targets in the same inoculum, the paired information can be used to remove this uncertainty.

Substituting eqn. (6) into eqn. (7) gives
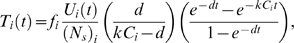
(8)removing the unknown quantity *c* = σ*N*(0) from the calculation, and where *d* = σ+δ. Thus *k* can be estimated using the measured values *T_i_*, *U_i_*, (*N_s_*)*_i_*, *f_i_* and *C_i_* together with the previous estimate of *d*.

An alternative is to work with the proportion of the pulsed targets in the spleen that have been killed, *p*(*t*)–
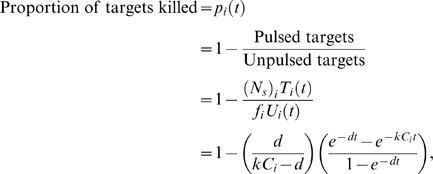
(9)where unpulsed target cell numbers are multiplied by the ratio *f* to correct for deviations from a 1:1 ratio of pulsed to unpulsed targets in the inoculum. Working with the proportion of targets killed makes visualising the raw data more straightforward, as we see below.

In either case, a two-step process is used to estimate *k*:

1. First estimate the loss rate of injected cells from the blood, *d* = σ+δ, using the unpulsed cell data alone. From eqn. (6), for each animal *i* the total number of unpulsed target cells in the spleen is
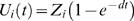
where *Z_i_* is proportional to *N_i_*(0). The parameter *d* can be estimated with a non-linear least squares fit to the data from all animals simultaneously and assuming *Z_i_* = *Z* is the same (and unknown) for all animals.
2. Use this estimate of *d* to generate point estimates of *k_NP_* and *k_GP_* using either equation (8) or (9).

Confidence intervals can be generated by repeating steps 1 and 2 on resampled datasets to generate empirical (bootstrap) distributions of all three parameter estimates (*d*, *k_NP_* and *k_GP_*).

### Transforming the data

Ideally the data should be transformed such that the errors are at least approximately normally distributed, justifying a least-squares fitting approach. Since the influx and loss of targets from the spleen are modelled as exponential processes, an obvious approach is to fit the logarithm of the target cell frequencies. However, these frequencies are measured by FACS analysis and at late timepoints when frequencies are low they are subject to increasing fractional error. In particular, we do not expect the assumption of constant error variance to hold on a logarithmic scale. For this reason we argue that it is incorrect to fit to either the logarithm or the logit of target cell frequencies (the logit function being approximately equal to the logarithm for small arguments). A reasonable alternative is to transform all cell frequency measurements using the arcsine-square-root [18], and the results we present here are generated using this data transformation. However, parameter estimates do not differ substantially when we fit directly to the untransformed cell frequencies or proportions.

### Selecting an estimation method

Some of the uncertainty in *k* comes from variability in the injected cell numbers *N*(0). We see from eqns (8) and (9) that *N*(0) does not appear explicitly in the second step of our revised estimating procedures. However, variation in *N*(0) generates uncertainty in the estimate of *d* in the first step, which propagates into the second step. To investigate whether this could introduce a bias in the estimate of *k*, we took a Monte Carlo approach. First we analysed the original data to estimate a distribution for the injected cell numbers *N*(0), and then generated artificial datasets using this distribution. We have
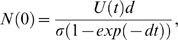
(10)where *U*(*t*) is measured, and *d* and *c* = *σN*_0_ were previously estimated Regoes, Barber, Ahmed, and Antia (*d* = 0.021, *c* = 6.6×10^3^). We constrain estimates of *σ* using the relations
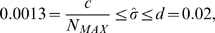
where *N_MAX_* is the approximate number of cells in each injection and so is an upper bound on the actual transferred number *N*(0). We used a value *σˆ* = 0.01, and then estimated *N*(0) for each animal using the observed values *U*(*t*) and eqn. (10). *N*(0) was well described by a lognormal distribution with log(mean) = 13.3 and log(sd) = 0.61. We then generated an artificial dataset by drawing a value *N*(0)*_i_* from this distribution and a value of *C_i_* from the empirical distribution of CTL frequencies, and using these numbers to generate *U*(*t_i_*) and *T*(*t_i_*) with eqns (6) and (7), using the original estimate *k* = 1.33 for NP targets killed by effector CTL. Replicates were run to generate a cohort of simulated animals and this dataset was used with the two-step estimation procedure to re-estimate *k*. We show the results of fitting the model with both methods in Table 1, as well as the results using the original method [13].

**Table 1:** Using a Monte Carlo approach to examine bias in the estimation methods.

Method	*k*	Std. error	95% CI
Original (Regoes et al.,PNAS 2007)	1.554	0.00720	(1.540, 1.568)
Direct fitting of target cell frequencies	1.354	0.00094	(1.353, 1.356)
Fitting the proportion of targets killed	1.332	0.00057	(1.331, 1.334)

Using 50000 simulated datasets each of 100 datapoints, we calculated the mean, standard error and 95% confidence intervals for the estimated killing rate *k* using the ‘true’ value *k* = 1.33 and using the arcsine-square root transformation.

The discrepancies between all estimates and the true value *k* = 1.33 were all significant (*t*-test, *p*<0.001). On the basis of this analysis we show fits to the proportion of targets killed, since it provides our least biased estimator.

Note that eqn. (9) can easily be solved numerically to obtain direct estimates of *k* for each animal. However, in this procedure the parameters governing target cell influx (*σ* and *δ*) are taken to be identical for each animal but the CTL killing rate *k* is assumed to be variable, which we feel is an unreasonable assumption.

## Results

### Revised estimates of killing rates with the basic model

**Figure 2:**
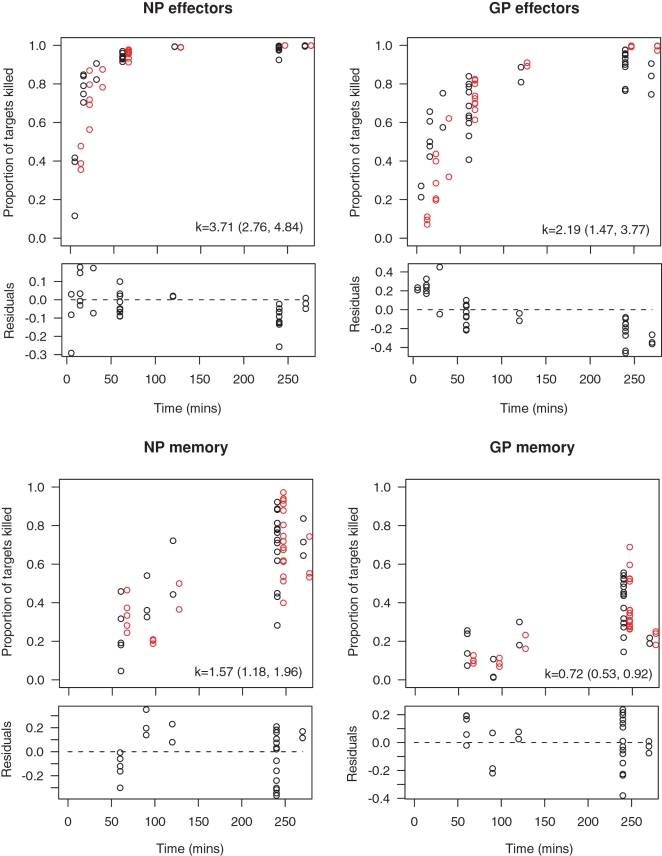
The fits (square panels) of the basic killing model to the data using the proportion of GP and NP-pulsed target cells that are killed over time, by effector CTL (upper panels) and memory CTL (lower panels) in the spleen. In the square panels the black open circles show the measured proportion of targets killed, and the fitted values for each animal are shown offset to the right in red open circles. The residuals after arcsin square-root transformation are shown below each fit in the rectangular panels. Best fit estimates of *k* in units of min^−1^ are shown in each panel, with 95% confidence intervals.

**Figure 3:**
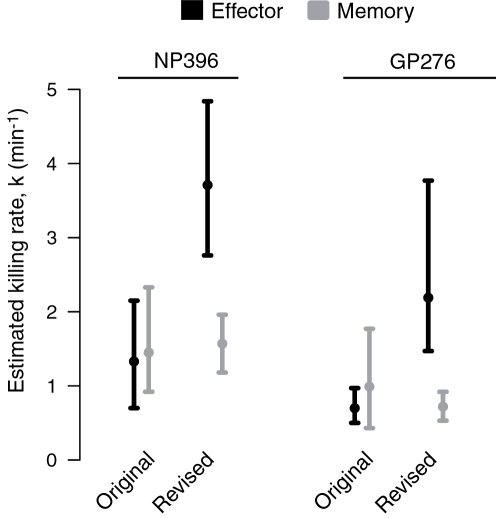
Estimates of the killing rate with 95% confidence intervals using the original procedure [13] and the revised method. This represents the data presented in Table 2.

Compared to the original estimates, the killing rates of NP396 and GP276-pulsed targets by effector CTL are both substantially increased, while memory cell killing rates are comparable to the originals. The raw data and fits are shown in Figure 2. Estimates of *k* are summarized in Table 2 and Figure 3.

**Table 2:** Original and revised estimates of killing rates, using paired estimates of pulsed and unpulsed targets.

		Original	Revised
Effector	*k*_NP_	**1.33** (0.70,2.15)	**3.71** (2.76,4.84)
	*k*_GP_	**0.70** (0.50,0.97)	**2.19** (1.47,3.77)
Memory	*k*_NP_	**1.45** (0.92,2.33)	**1.57** (1.18,1.96)
	*k*_GP_	**0.99** (0.43,1.77)	**0.72** (0.53,0.92)

Confidence intervals are shown in parentheses and were calculated using the adjusted percentile method with 1000 (original results) or 2000 (revised estimates) bootstrap replicates.

For all data, the estimates of *k* are essentially unchanged if we fit directly to the untransformed rather than arcsin square-root transformed proportions.

### Discrepancies between the fits and data

This new approach allows clearer visualisation of the fits to the data (Figure 2). In three of the four cases, the fits are reasonable as assessed by the distribution of the residuals over time. However, for GP-specific effector cells, the proportion of targets killed is overestimated by the basic model at late timepoints; there is a trend in the residuals over time, and the data appear to saturate at less than 100% of targets. There are several potential explanations for this.

#### 1. Refractory targets

A proportion *q* of the GP-pulsed cells entering the spleen are susceptible to killing, and the remainder (1-*q*) are effectively “invisible”. With this modification to the model, the proportion of targets killed is now simply
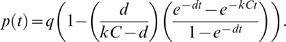
(11)This extended model for GP effectors improves the fit significantly (*p*<10^−3^, F-test), provides an increased estimate of *k_GP_* of 5.32 (2.78, 8.50), and predicts that around 10% of GP targets are effectively invisible to CTL in the spleen (*q* = 0.87 (0.81, 0.92)).

There are at least two ways in which cells might be refractory. They can be invisible to detection (either by presenting insufficient peptide to be recognized by CTL, or by migrating to areas of the spleen not accessible to CTL), or be resistant to killing. However, these hypotheses are perhaps unlikely on the grounds that they must be compatible with the remainder of the data. For example, if there are areas of the spleen that are inaccessible to CTL, or a proportion of targets are resistant to killing, then we expect a similar proportion of targets to evade NP-specific effector CTL, which is not observed (Figure 2, upper L panel). Also, since all targets were pulsed with high doses of peptide, we do not expect any targets to present peptide at levels below that required for CTL recognition. However, this could be further tested by injecting with targets pulsed with even larger amounts of peptide.

#### 2. Epitope decay

The GP epitopes are progressively lost from target cells. In H-2b mice the GP276 epitope binds significantly more weakly to MHC class I than NP396, with a 10-fold higher dissociation rate [19]. If we assume that as a result of epitope decay the GP effector killing rate *k_GP_* falls exponentially with time as *k_GP_* = *k*_0_ *exp*(−*μt*), we estimate *k*_0_ = 4.05 (3.02, 5.76) and *μ* = 7.72 (6.16, 9.36)×10^−3^ min^−1^ for killing by GP-specific effector CTL. This implies that the half life of the decay of *k* is around 90 minutes. This model is also unlikely, however, since while it improves the fit for GP effector cells (*p*<10^−8^, F-test), it fails to improve the fit for GP memory. Indeed, the loss of the GP276 epitope from target cells should have a greater impact on killing by memory CTL, since net rates of killing are slower due to lower CTL density. However, this model could be further tested by incubating GP-pulsed targets prior to injection. GP killing rates should then be further decreased.

#### 3. CTL exhaustion

When a CTL kills a target cell it is lost from the pool of functional effector CTL and has to ‘recharge’. If CTL are unable to recharge during the short time course of the experiment, functional killers are then simply lost at rate equal to the net rate of target cell loss, *kCT*. This model contains the same parameters as the original and yields slightly increased estimates of the GP killing rates, but does not reduce the residual sum of squares significantly; *k_GP_* (effector) = 2.30, ΔAIC = 2.96; *k_GP_* (memory) = 1.03, ΔAIC = 3.20. Despite this, the CTL exhaustion model could be tested further by injecting a second (differently labelled) cohort of pulsed and unpulsed targets soon after the first, and determining whether the second cohort are killed more slowly than the first.

## Discussion

Measuring CTL killing rates with this *in vivo* assay requires not only modeling killing itself but also the flow of targets into the spleen. We have improved our previous analysis by removing one major source of uncertainty (variation in the number of targets transferred intravenously) and using the natural pairing of measurements to make fuller use of the information contained in the data. In contrast to previous estimates we now predict that effectors survey and kill approximately twice as rapidly as memory cells. Further, in both effector and memory responses CTL specific for the immunodominant NP396 epitope kill their targets approximately twice as fast as CTL specific for the subdominant GP276.

These estimates of CTL killing rates complement the information gained from other methods, and in particular in two photon intravital microscopy. Recent advances in this area allow the direct visualization of killing in small regions of tissue or a lymph node (e.g. [20], [21]). This technique allowed the measurement of the times spent in delivering the lethal hits to pulsed targets as well as the time spent browsing pulsed and unpulsed targets. Unfortunately the study by Mempel *et al.* [20] does not provide information regarding the time unbound CTL take to locate and attach to potential targets. This prevents direct comparisons with our estimate of the rate *k*, which is the inverse of the mean time to locate and survey targets. Mempel *et al.* also show that CTL effector function can be limited by regulatory T cells [20]. It is therefore possible that different levels of inhibition acting on CTL specific for different LCMV epitopes could influence CTL killing rates and that our estimates are not the ‘true’ killing rates of these populations. Clearly, combining our assays with direct visualization would be a powerful test allowing for the validation of both methods.

Despite the improvements to our method we have described, a small discrepancy between one of the four datasets and the fit (for the GP effector data) suggests that the killing model could be further refined. There are also other potential sources of error, both in the assay and the models.

First, our estimate of *k* assumes that the ratio of pulsed to unpulsed cells migrating into the spleen during the experiments is identical to the ratio in the initial inoculum. This assumption might be violated if there is differential sequestration of pulsed and unpulsed targets cells in other tissues (such as the lungs). If cells exposed to CTL in other organs in the body recirculate into the blood, this will lead to enrichment of unpulsed targets in the blood and overestimation of killing rates in the spleen. Conversely, if recirculation of cells exposed to CTL can be neglected, our estimate of the killing in the spleen is unaffected. The importance of extra-splenic killing and recirculation could be tested with non-destructive sampling of the blood immediately after transfer (that is, over the short timescale during which cells are migrating into the spleen) to test that the ratio of pulsed to unpulsed targets in blood is preserved.

A second potential problem is the assumption that killing can be described by a mass-action term. Our models assume a killing rate of *kCT* where C and T are the CTL and target cell frequencies in the spleen, respectively. This is expected to hold for well-mixed populations at intermediate C:T ratios. When the C:T ratio is very low ( = 1) the net rate of killing is limited not only by the encounter rate of CTL and targets, as assumed by mass-action, but also by CTL recycling and/or the ‘dwell time’ taken for a single CTL to browse a potential target cell, deliver the cytolytic granules and detach from it. At high CTL densities and high C:T ratios, multiple CTL may bind to a single target and so mass-action may also break down. For GP memory cells the mean C:T (or ‘effector:target’) ratio over all timepoints was 1.46 (1.09, 2.08) and for NP memory it was 4.92 (3.43, 7.46), and so we might expect mass action to hold. For effectors, C:T was 917 (1350, 3040) for NP and 129 (87, 193) for GP. Particularly for the effector populations, then, estimates of *k* might be improved further by introducing the possibility of pulsed targets being hit by multiple CTL.

A third potential issue is that we identified specific CTL using tetramer staining only. It is possible that only a proportion of these cells express effector molecules such as perforin and granzyme and are capable of killing. Clearly, overestimating the number of functional CTL will underestimate killing rates. However, the relationship between killing *in vivo* and the expression levels of these molecules has not been definitively established. For example, exhausted CTL express significantly higher levels of Granzyme B than memory cells [22] and yet are unable to kill, and CTL can kill without perforin [23]. The method we propose smoothes over any heterogeneity in the epitope-specific CTL response and provides an average efficiency of killing for each specificity.

Finally, we note an important difference between our approach and those used in quantitative studies of cell lysis or virus dynamics. For example, in HIV infection, the analysis of the decay of circulating virus in the blood directly after treatment with anti-viral drugs allows the extraction of important biological parameters such as infected cell lifetime and the half life of free virus [24]. With the assay and models we discuss here, pulsed/unpulsed target cell numbers in the spleen should asymptotically approach exponential decay with half life ln(2)/(*kC*) once influx is complete. However, by this stage target cell numbers are small and difficult to measure accurately, and measurements may be further complicated by efflux from the spleen. For these reasons, maximum information for the estimation of *k* perhaps comes from early or intermediate timepoints when both influx and killing must be considered.

Our work stresses how a close collaborations between experimental and theoretical immunologists is vital in order to measure important quantities such as how fast CTL can find and kill target cells.
